# Rapid High-Yield Production of Functional SARS-CoV-2 Receptor Binding Domain by Viral and Non-Viral Transient Expression for Pre-Clinical Evaluation

**DOI:** 10.3390/vaccines8040654

**Published:** 2020-11-04

**Authors:** Omar Farnós, Alina Venereo-Sánchez, Xingge Xu, Cindy Chan, Shantoshini Dash, Hanan Chaabane, Janelle Sauvageau, Fouad Brahimi, Uri Saragovi, Denis Leclerc, Amine A. Kamen

**Affiliations:** 1 Viral Vectors and Vaccines Bioprocessing Group, Department of Bioengineering, McGill University, Montréal, QC H3A 0E9, Canada; omar.farnosvillar@mcgill.ca (O.F.); alina.venereo-sanchez@mcgill.ca (A.V.-S.); xingge.xu@mail.mcgill.ca (X.X.); cindypsc14@gmail.com (C.C.); shantoshini.dash@mail.mcgill.ca (S.D.); hanan.chaabane@gmail.com (H.C.); 2 Human Health Therapeutics, National Research Council of Canada, Ottawa, ON K1A 0R6, Canada; Janelle.Sauvageau@nrc-cnrc.gc.ca; 3 Lady Davis Institute-Jewish General Hospital, McGill University, Montreal, QC H3T 1E2, Canada; fouad.brahimi@yahoo.fr (F.B.); uri.saragovi@mcgill.ca (U.S.); 4 Department of Pharmacology, Department of Ophthalmology and Visual Science, McGill University, Montréal, QC H3A 1A3, Canada; 5 Département de Microbiologie-Infectiologie et d’immunologie, Faculté de Médecine, Université Laval, Québec City, QC G1V 0A6, Canada; Denis.Leclerc@crchudequebec.ulaval.ca

**Keywords:** SARS-CoV-2 Spike Receptor Binding Domain, HEK293SF suspension cells, vaccine bioprocess, RBD, COVID-19 vaccine, bioreactor

## Abstract

Vaccine design strategies against severe acute respiratory syndrome coronavirus 2 (SARS-CoV-2) are focused on the Spike protein or its subunits as the main antigen target of neutralizing antibodies. In this work, we propose rapid production methods of an extended segment of the Spike Receptor Binding Domain (RBD) in HEK293SF cells cultured in suspension, in serum-free media, as a major component of a COVID-19 subunit vaccine under development. The expression of RBD, engineered with a sortase-recognition motif for protein-based carrier coupling, was achieved at high yields by plasmid transient transfection or human type-5-adenoviral infection of the cells, in a period of only two and three weeks, respectively. Both production methods were evaluated in 3L-controlled bioreactors with upstream and downstream bioprocess improvements, resulting in a product recovery with over 95% purity. Adenoviral infection led to over 100 µg/mL of RBD in culture supernatants, which was around 7-fold higher than levels obtained in transfected cultures. The monosaccharide and sialic acid content was similar in the RBD protein from the two production approaches. It also exhibited a proper conformational structure as recognized by monoclonal antibodies directed against key native Spike epitopes. Efficient direct binding to ACE2 was also demonstrated at similar levels in RBD obtained from both methods and from different production lots. Overall, we provide bioprocess-related data for the rapid, scalable manufacturing of low cost RBD based vaccines against SARS-CoV-2, with the added value of making a functional antigen available to support further research on uncovering mechanisms of virus binding and entry as well as screening for potential COVID-19 therapeutics.

## 1. Introduction

Severe acute respiratory syndrome coronavirus (SARS-CoV) and the Middle East respiratory syndrome coronavirus (MERS-CoV) have caused the relatively recent SARS and MERS epidemics, respectively. To date, no vaccines or effective antivirals exist to combat these diseases. In contrast to SARS-CoV (2002–2004), severe acute respiratory syndrome coronavirus 2 (SARS-CoV-2), causing coronavirus disease 2019 (COVID-19) has been shown to be far more threatening to humans [1]. As of 25 September 2020, COVID-19 has spread worldwide, with 32,509,047 confirmed cases, 989,275 associated deaths [2] and no signs for a decreasing trend in the number of the daily new cases reported. Elderly people and individuals with underlying health conditions constitute the population at higher risk for a serious, critical course of the disease [3].

Following the influenza pandemic H1N1 in 2009 (swine flu), 2010-20 was declared the decade of vaccines with the goal of investing in preparedness to face emerging and re-emerging infectious diseases [4]. It resulted in an international consensus calling for a coordinated intergovernmental plan to develop and deploy new vaccines to prevent future epidemics [5]. However, despite repeated public health crises such as the African outbreaks of Ebola since 2014, the COVID-19 pandemic found the world in a state of global unpreparedness [6]. Yet, previous developments in technology platforms, building on previous advances in recombinant technologies and cell culture processing methodologies are available for a variety of vaccine manufacturing alternatives. Vaccination remains the most effective means to prevent and contain infectious diseases such as COVID-19. Many different types of candidates are currently in development including ones based on replicating and non-replicating viral vectors, DNA, mRNA, subunit proteins, virus-like particles (VLP) as well as inactivated and attenuated vaccines [7,8]. Many of the vaccines currently in pre-clinical or clinical phases use antigen design strategies and production processes that had been developed for other diseases, strongly supporting the concept of investing in technology platforms. Due to the complex biology of SARS-CoV-2 and the subsequent response of the human immune system, it is not possible to predict which class of vaccine will provide the best immunity against the virus. Given the magnitude of this pandemic and the probability to be further affected by high-impact infectious diseases, investments in building long-lasting capacities and the accelerated development of any type of platform technologies to manufacture safer, protective and cost-effective vaccines are urgently needed.

Coronaviruses’ entry into the host cell is the first and most important step in the viral pathogenesis [9]. For SARS-CoV-2, a fragment from the Spike (S) protein S1 subunit, the receptor binding domain (RBD), is required for binding to the peptidase domain of angiotensin converting enzyme 2 (ACE2), which is widely expressed in various human organs including lungs, heart, brain and kidney [10]. The S protein is the major structural protein displayed as a trimer on the surface of the SARS-CoV-2; it carries many B cell and T cell epitopes and is the target (specifically the RBD) of neutralizing antibodies that provide protection against viral infection [11,12]. In this work, for research and applied multipurpose objectives, the RBD has been rapidly expressed and purified using the human cell line HEK293SF cultured in suspension and serum-free media in order to ensure the proper protein folding and similar post-translational modifications (glycosylation) to the native antigen. Expression strategies were approached by viral and non-viral rapid expression systems and the scalability of these simple production processes was assessed in paralleled bioreactor runs. Process development steps were included, seeking for increased production yield and recovery of the antigen in a shortest period of time. The recombinant products were characterized and compared, including their monosaccharide composition and affinity binding to ACE2. RBD produced from the processes described is intended to be used in the manufacturing of COVID-19 vaccine candidates under different conceptual strategies and is also an important tool for the study of virus-host interactions and the selection of potential therapeutic/blocking agents of SARS-CoV-2. 

## 2. Materials and Methods

### 2.1. Plasmids

The Receptor Binding Domain (RBD) sequence from the NCBI Reference Sequence for SARS-CoV-2 Accession no. NC_045512.2 was used for the chemical synthesis (Topgenetech Technologies, St-Laurent, Quebec, QC, Canada) of the S gene fragment selected for expression using an optimized mammalian cells expression plasmid (pADCMV5) [13]. In this vector, expression of the gene of interest, which was codon optimized for humans, is driven by the human CMV promoter and the rabbit beta-globin polyA signal. The sequence NC_045512.2 has 100% identity with the sequence MN908947.3, which was the first SARS-CoV-2 genome sequence (Wuhan-Hu-1) successfully obtained and submitted to GenBank. The extended RBD sequence selected covered amino acids 331 to 591 in SARS-CoV-2. Two different signal peptides were used at the N-terminal of the RBD in two different constructions: the 20-mer native signal peptide of the Spike protein and the 35-mer secretion signal of the human tissue plasminogen activator (hTPA). A 6 x His tag was placed downstream of the signal peptide, at the N-terminal of RBD, followed by an enterokinase recognition cleavage site and the RBD coding sequence. The protein also carries a coupling domain consisting of a six amino acids length sortase recognition site at its C-terminal [14].

Both sequence variants of the SARS-CoV-2 RBD (with the different signal peptides) were inserted into the pADCMV5 vector between PmeI and BamHI restriction sites. The resultant plasmids were designated as pNative_SP-RBD-His-SrtA and pTPA_SP-RBD-His-SrtA. These expression plasmids were amplified and purified from competent *E. coli* cells (New England Biolabs, Whitby, ON, Canada) using the Qiagen Plasmid Giga Kit (Qiagen, Toronto, ON, Canada) for purification of up to 10 mg of transfection-grade plasmid DNA.

### 2.2. Adenovirus Constructs and Seed-Stock Generation

The two SARS-CoV-2 RBD coding sequences described were also inserted in KpnI/HindIII restriction sites of the adenoviral transfer vector pShuttle-Cytomegalovirus (CMV) for the generation of replication-deficient human adenoviruses type 5 (Agilent Technologies, Saint-Laurent, QC, Canada) in which the selected RBD gene fragment is expressed, respectively, under the native Spike signal peptide or the hTPA. The pShuttle vector contains the human CMV promoter, followed by the multiple cloning site and the SV40 polyadenylation signal for transcription termination. These vectors were transformed in the BJ5183 *E. coli* strain which contains the pAdEasy plasmid (Agilent Technologies) carrying the adenoviral genome deleted for the E1 and E3 regions. Adenoviral vectors were obtained by recombination of the adenoviral genome and the shuttle vectors carrying the RBD. The shuttle vectors, as well as the final plasmids carrying the foreign genes, were verified by restriction endonuclease analysis and sequencing. A final digestion step using Pac I enzyme allowed transfection into the E1-complementing HEK293A cells for the rescue of adenoviral-assembled particles after 5–7 days in culture. Primary viral stocks were prepared and stored at −80 °C after a single amplification step in HEK293SF. These seed-stocks were further assessed for infectivity and expression of the antigens. The final adenoviral vectors generated were designated as Ad-native_SP-RBD-His-SrtA and Ad-hTPA_SP-RBD-His-SrtA. Determinations of infectious titers (IVP/mL) were performed using HEK293A in 96-well plates as described before [15] using HEK293A cells. The Median Tissue Culture Infectious Dose (TCID_50_) per mL value was calculated according to the Spearman–Kärber method [16]. A reference control was run in every determination to ensure an acceptable reproducibility of the assay.

### 2.3. Cells Lines and Culture Media

HEK293A adherent cells were kindly provided by the National Research Council of Canada (Montréal, QC, Canada). They were used with transfection protocols aimed at the rescue of the adenoviral vectors as described above. HEK293A cells were also used for the TCID_50_ assay. HEK293A cells were maintained in cell culture dishes in a static humidified incubator at 5% CO_2_ and 37 °C in Dulbecco’s Modified Eagles Medium (DMEM, Wisent, Saint-Jean-Baptiste, QC, Canada), supplemented with 10% Fetal Bovine Serum (FBS, Gibco, Gaithersburg, MD, USA). Cells were passaged twice a week, and detached at confluence using Trypsin (Millipore Sigma, Oakville, ON, Canada). After centrifugation at 400 *g* for 5 min, the cells were resuspended in fresh medium and seeded at 1:10 dilution.

The HEK293 serum-free cell line used for RBD production is derived from HEK293SF (clone 293SF-3F6) suspension cells originating from a GMP-grade master cell bank [17]. These cells were used for plasmid DNA transfection for rapid production of secreted RBD in the culture supernatant. They were also used for amplification of the RBD-expressing adenoviral type 5 vectors, for virus primary stock preparation, and for Ad5-mediated RBD production in shake flasks and controlled 3L-bioreactors. HEK293SF cells were grown either in disposable polycarbonate vented-cap shake flasks (Corning, Tewksbury, MD, USA) or bioreactors. For maintenance, cells were passaged twice per week by diluting to 2.5 × 10^5^ viable cells per mL in fresh medium. They were grown either in HyClone HyCell TransFx-H medium (Cytiva, Marlborough, MA, USA) or Xell AG HEK-GM medium (Xell AG, Bielefeld, Germany). Both are chemically defined, animal-component-free, with no antibiotics added. HyCell TransFx-H was supplemented with 6 mM Gibco GlutaMAX (Fisher Scientific, Saint-Laurent, QC, Canada) and 0.1% Kolliphor poloxamer 188 (MilliporeSigma, Oakville, ON, Canada). When grown in HEK-GM, they were supplemented only with 6 mM Gibco GlutaMAX. In all cases, cell counts and cell viability were determined in a Vi-CELL XR cell counter (Beckman Coulter Life Sciences, Brea, CA, USA).

### 2.4. Production of the Receptor Binding Domain in Shake-Flasks Cultivation Experiments 

The media HyCellTransFx-H and HEK-GM were used for plasmid DNA transfection and adenoviral-mediated production of RBD, respectively, in 250 mL shake flask experiments with 40 mL of initial working volume. An orbital shaker platform (Infor’s HT, Montréal, QC, Canada) was operated at 110 revolutions per minute (rpm) in conditions of 80% humidity, 5% CO_2_, and 37 °C.

For cultures transfected with the pNative_SP-RBD-His-SrtA and pTPA_SP-RBD-His-SrtA expression plasmids, optimization assays were preliminarily conducted in this work until establishing the most productive transfection conditions, consisting of 1 ug of plasmid DNA per mL of culture medium, using Polyethylenimine (PEI) (Linear, MW 25,000, PolyScience, Warrington, PA, USA) at a mass ratio of DNA–PEI of 1:3. Cultures were seeded at a cell density of 0.25 × 10^6^ cells/mL, and the transient transfection was performed when cells reached 1 × 10^6^ cells/mL. Samples from culture supernatants were taken at 24, 48 and 72 h post-transfection, respectively, and transfections were stopped at 72 h post-transfection.

For adenoviral infection of the HEK293SF cells, shake-flasks experiments were conducted to first assess the performances of culture media and supplements in the virus-mediated production of RBD. For the subsequent evaluation of the RBD expression in culture supernatants, both HyCellTransFx-H and HEK-GM media were used with and without addition of supplements before and during infection, for the subsequent evaluation of the RBD expression in culture supernatants. Supplementing the HEK-GM basal media consisted of a bolus addition of HEK-FS (Xell AG, Bielefeld, Germany) supplemented with 4 mM GlutaMAX, starting from day 1 of the culture at 5% (*v/v*), every second day, until the end of the culture. Supplementing the HyCellTransFx medium was carried out with Cell Boost 5 (Cytiva, Marlborough, MA, USA) containing 4 mM GlutaMAX, following an identical scheme until the end of the culture. Infections with the Ad-native_SP-RBD-His-SrtA and Ad-hTPA_SP-RBD-His-SrtA adenoviruses were evaluated at 1 and 2 × 10^6^ cells/mL with the media described. Cultures were stopped at about 40 h post-infection, when cell viability decreased to approximately 70%. Infection experiments at low (0.1) and high MOI (5) were also performed in cultures infected at 5 × 10^6^ cells/mL using both media, followed by determination of RBD expression levels secreted to the culture medium. All the experiments were performed in triplicate.

### 2.5. Production of the Receptor Binding Domain in 3L-Controlled Bioreactors

Plasmid DNA transfection and adenovirus-mediated production of RBD were evaluated in 3 L bioreactors (Applikon Biotechnology, Delft, The Netherlands) using the pTPA_SP-RBD-His-SrtA and the Ad-hTPA_SP-RBD-His-SrtA genetic constructions, respectively. The 3 L bioreactor (working volume 2.7 L) was equipped with a double marine impeller and a capacitance probe (Aber instruments Ltd., Aberystwyth, UK) to measure cellular biomass. The reactors were seeded at a viable cell density ranging between 0.25 and 0.5 × 10^6^ cells/mL in HyCellTransFx-H or Xell AG HEK-GM medium. For transfection, cells were grown in HyCellTransFx-H until reaching cell densities of 1 × 10^6^ cells/mL, in which they were transfected as previously described and monitored for 72 h until the production run was stopped.

For adenoviral infection, cells were grown until reaching approximately 2 × 10^6^ cells/mL, and infected with a MOI of 5. Prior to infection, the culture was supplemented with a 5% bolus feed starting at a viable cell count of approximately 1 × 10^6^ cells/mL. The feeding was maintained as previously described. For both independent runs, the bioreactor control unit maintained controlled conditions of DO concentration at 40% air saturation by continuous surface aeration of 12.5 mL/min and injection of pure oxygen when required. The pH was set to 7.15 and regulated by the injection of CO_2_ into the headspace or the addition of NaHCO_3_ (90 g/L) (Millipore Sigma). Agitation was kept at 100 rpm and increased to 120 rpm in the last 24 h of culture, to circumvent the formation of cell aggregates. Monitoring of the infected culture during the growth and production phases was achieved through the analysis of capacitance values (uF/cm) as an indication of the total biovolume of the culture, using the capacitance probe. For transfected cells, harvest was done at 72 h post-transfection whereas in virus-mediated productions, production runs were halted when cell viability reached around 70%, at approximately 40 hpi. Samples were taken every 24 h to measure cell density and viability.

### 2.6. Downstream Processing

#### 2.6.1. Cells Harvest, Culture Supernatant Clarification and Medium Exchange

The harvested material from the 3L bioreactor runs was centrifuged at 1000× *g* for 10 min to separate the cells from the culture supernatant. After centrifugation, the culture medium from adenovirus infected cells was subjected to tangential flow filtration (TFF), implemented in a Biosafety Cabinet Level II using a 300 KDa hollow fiber filtering device (Repligen, Waltham, MA, USA) in order to separate the adenovirus- containing retentate from the fraction containing the recombinant RBD. The permeate fraction from the latter step and the supernatant from plasmid transfected cells were first filtered through 0.2 um using bottle tap vacuum filters (Thermo Fisher, Waltham, MA, USA). The culture supernatants were buffer exchanged using 10,000 Da Amicon Ultra-15 centrifugal tubes (Millipore Sigma) by subsequently loading 15 mL of culture medium and centrifuging at 4000× *g* until approximately 0.5 mL of retention volume was observed. For large volumes of culture supernatant, TFF was implemented, by using a hollow fiber filter with the same cut-off. Both media were exchanged by equilibrium buffer (20 mM sodium phosphate buffer, 20 mM imidazole, pH 7.4). These supernatants were collected, pooled and loaded for immobilized metal ion affinity chromatography (IMAC). Independent lots with volumes ranging from 100 to 650 mL were prepared from each bioreactor until the processing of the entire volume collected was completed.

#### 2.6.2. Immobilized Metal ion Affinity Chromatography

The recombinant RBD was purified using HisTrap HP 5 mL affinity columns with AKTA^TM^ Avant 25 system (Cytiva). A same loading-washing-eluting procedure was adapted for both transient transfection derived medium and adenovirus infection medium. Buffer exchanged samples of about 650 mL medium were loaded to the column at a flow rate of 2.5 mL/min. After loading, the column was washed with equilibrium buffer at 5 mL/min until UV 280 nm absorbance signal returned to the baseline. Then, the column was washed with buffer (20 mM sodium phosphate buffer, 60 mM imidazole, and 500 mM NaCl, pH 7.4) to remove non-specifically bound contaminants. The RBD was eluted in a single step elution performed at 2.5 mL/min with imidazole concentration of 150 mM. A final wash step using 20 mM sodium phosphate, 500 mM imidazole, 500 mM NaCl, pH 7.4 was conducted, to elute all residual proteins and proceed to re-equilibrate the column before the next run. The elution pools were buffer exchanged one last time using a storage buffer (1× PBS buffer, 2 mM MgCl_2_, and 2% sucrose, pH 7.2) and 10,000 Da cut-off centrifugal Amicon tubes.

### 2.7. Analytical Assays for Characterization of the Recombinant Antigens

#### 2.7.1. Protein Quantification

Total protein from the samples obtained in the different downstream process (DSP) steps was quantified by DC protein assay kits (Bio-Rad, Hercules, CA, USA). High concentration samples were diluted with PBS 1X to fit into the bovine serum albumin (Bio-Rad) standard curve, ranging from 0 to 250 µg/mL. The standards and unknown samples were processed in Corning™ Polystyrene 96-Well Microplates (Fisher Scientific Inc., Mississauga, ON, Canada) and read with a microplate reader (Synergy HTX Multi-Mode Reader, Biotek Instruments, Winooski, VT, USA) at 750 nm.

#### 2.7.2. SDS-PAGE and Western Blot Analyses

Two monoclonal antibodies were carefully selected from the recent literature and used for varied purposes: the monoclonal antibody HC2001 and the polyclonal anti-RBD (Cat: PA5-81795) were used for Western blot analysis. The conformation-dependent CR3022 was only utilized for RBD quantification in ELISA. Samples from DSP were separated by SDS-PAGE, fixed and analyzed by silver staining according to manufacturer’s instructions for the Silver Stain Plus Kit (Bio-Rad, USA). For gel loading, 1 µg of total protein or maximum volumes of 18 ul per well of samples diluted in 4x Laemmli Sample Buffer (Bio-Rad) were loaded onto the gels. As reducing agent, 50 mM dithiothreitol was added, followed by denaturation at 95 °C for 5 min. Samples containing 0.5 µg of RBD protein (Cat. No. Z03483, SARS-CoV-2 Spike protein RBD, His Tag, GenScript, Piscataway, NJ, USA) were loaded for comparative purposes at the initial stages of the work. Electrophoreses were run in Tris/glycine-SDS running buffer under 120 V constant voltage. Samples were alternatively run under non-reducing conditions for evaluation of potential presence of RBD multimeric structures. Parallel gels were also transferred onto nitrocellulose membranes for Western blot analyses.

The analysis by western blot was conducted as follows, with three combinations of antibodies; (i) 6X-His Tag Monoclonal Antibody (His.H8) (Thermo Fisher, Waltham, MA, USA) as primary antibody at a dilution 1:1000 and Goat Anti-Mouse IgG-HRP (Jackson ImmunoResearch, West Grove, PA, USA) as secondary antibody at a dilution 1:10,000, (ii) monoclonal antibody anti-SARS-CoV2 S1 HC2001 (GenScript, Piscataway, NJ, USA) as primary antibody at a dilution 1:1000 and anti-human IgG (H + L) HRP Conjugate (Promega, Madison, WI, USA) as secondary antibody at a dilution 1:2500, (iii) polyclonal antibody anti-SARS-CoV2 Spike Subunit 1 (Thermo Fisher) as primary antibody at a dilution 1:2000 and Peroxidase-AffiniPure Goat Anti-Rabbit IgG (H + L) (Jackson ImmunoResearch) as the secondary antibody at a dilution 1:1000. In western blot analyses, for comparative purposes, equal amounts of total proteins were applied onto the wells, according to DC protein assay (Bio-Rad) quantifications. In all cases, a TransBlot^TM^ semi-dry transfer (BioRad) equipment was used, followed by blocking the membranes with 5% skim milk. The primary antibody was incubated for 1 h followed by three washes with PBST (PBS–0.1% Tween-20) and the secondary antibody was added for 1 h and subsequently washed. All antibodies were diluted in PBST + skim milk 5%. Protein bands were visualized with Clarity™ Western ECL Substrate (Bio-Rad, Hercules, CA, USA).

#### 2.7.3. Protein Quantification by Enzyme Linked Immunosorbent Assay (ELISA)

A competitive ELISA was adapted and optimized for quantification of the RBD antigen produced in HEK293SF cells in suspension, following principles and guidelines for the quantification of SARS-CoV-2 antigens [18]. The conformation-dependent CR3022 monoclonal antibody (Absolute Antibody, Boston, MA, USA) was used as detection antibody in one incubation step with serial dilutions of test samples of unknown concentration and RBD protein of known concentration, used as standard. This antigen-antibody complex was added to ELISA plates and incubated for binding to previously immobilized RBD protein. The standard curves were constructed using the transient transfection-produced RBD protein quantified by densitometric analyses. A secondary HRP-conjugated antibody was used, followed by OD measurements at 450 nm (Synergy HTX Multi-Mode Reader, Biotek Instruments). Reference RBD or S1 of known concentrations were included for corroboration of the accuracy of measurements.

### 2.8. RBD Analysis of Oligosaccharides

#### 2.8.1. Deglycosylation with PNGase-F

The RBD was denatured at 100 °C for 10 min in glycoprotein denaturation buffer (New England Biolabs). Afterwards, it was cooled on ice and kept at room temperature. Two microliters of GlycoBuffer 2 (10X), 2 µL 10% Nonidet P-40 detergent (NP-40) and H_2_O were added for a final volume of 20 µL before PNGase F addition. PNGase F digestion was carried out at a ratio of 5 U per mg of protein and incubated for 1 h at 37 °C. Enzymatic deglycosylation was verified by western blot using the rabbit anti-S polyclonal antibody.

#### 2.8.2. Chemical and Enzymatic Hydrolysis

Sialic acid, fetuin and the Amicon ultra-4 3 kDa molecular weight (MW) cut off spin column were purchased and used in the experiment (Millipore Sigma). Monosaccharide standard mixture and 50% sodium hydroxide, electrochemical grade sodium acetate were purchased from Thermo Fisher Scientific Inc. (Mississauga, ON, Canada). Sialic acid was removed from RBD by enzymatic deglycosylation (neuraminidase digestion) using standard procedures (Thermo Fisher Scientific, Inc.) [19]. Each sample was digested in triplicate and results were extrapolated using a Neu5Ac standard curve. For other monosaccharides, chemical deglycosylation was utilized as follows: the bulk of sucrose was removed from the RBD formulation buffer by washing it through an Amicron ultra-4 3 kDa MW filter against PBS (5 × 4 mL). The RBD (25 µg) was hydrolysed in triplicates using TFA (3.6 M) for 2 h at 100 °C. The hydrolyzates were evaporated in a EZ-2.3 Elite Genevac evaporator and resuspended in dH_2_O (RBD 150 µL and Fetuin 50 µL) and spun (13 k, 2 min). The molecular ratios were calculated using the molecular weight of RBD = 35.0 kDa.

#### 2.8.3. High-Performance Anion-Exchange Chromatography with Pulsed Amperometric Detection (HPAEC-PAD)

Monosaccharide and sialic acid analyses were performed on a IC-6000 (Thermo Fisher Scientific Inc.) equipped with an electrochemical detector with a carbohydrate 4-potential waveform, a gold electrode and an Ag/AgCl reference electrode. The data were analyzed with Chromeleon 7. The monosaccharides and sialic acid were identified and quantified with a monosaccharide standard mixture and calibration curve. For monosaccharide analysis, samples were eluted (25 µL, RBD at 1:20 dil. and Fetuin at 1:10 dil. V:V) on a Dionex CarboPac PA20 column (3 × 150 mm, Thermo Scientific) preceded with a Borate trap column (4 × 50 mm) and an Amino Trap column (3 × 30 mm). The elution conditions were as follow: 10 mM NaOH step (12 min), 200 mM NaOH (10 min), and 10 mM NaOH step (18 min). For sialic acid analysis, samples were injected (25 µL, 1:10 dil. V:V) on a Dionex CarboPac PA20 column (3 × 150 mm, Thermo Scientific) preceded with a PA20 trap column (3 × 30 mm). Elution conditions were as follow: 70–300 mM sodium acetate in 100 mM NaOH (0–7.5 min), 300 mM sodium acetate in 100 mM NaOH (7.5–9.0 min), 300–70 mM sodium acetate in 100 mM NaOH (9.0–9.5 min), 70 mM sodium acetate in 100 mM NaOH (9.5–16.5 min).

### 2.9. RBD Binding to Angiotensin-Converting Enzyme 2

The flow cytometry assay was performed by standard procedures. HEK293 cells stably transfected with human ACE2 cDNA (Sino Biological, Wayne, PA, USA) were resuspended in 0.1 mL of flow cytometry buffer and incubated with the test proteins (RBD, to which a His-Tag was engineered at the N-terminal, produced by two manufacturing processes) or a standard S1 reference protein at 0.25 and 0.5 µg/mL for 20 min at 4 °C. The RBD samples from the transfection and infection approaches used were those from the final elutions in which RBD was detected with a purity over 95% by silver staining of SDS-PAGE gels. Cells expressing ACE2 were washed and incubated with an anti-His tag antibody conjugated to Alexa Fluor 488 (R&D Systems, Cedarlane, Canada) for 20 min at 4 °C. Cells were washed, resuspended in flow cytometry buffer and data acquired from the cytometer was analyzed using the FlowjoV9 software. As negative controls, no antibody (background fluorescence) or irrelevant mouse IgG were used. As positive control, an anti-human ACE2 monoclonal antibody was used (Cat: AHA007, Sanyou Biopharmaceuticals, Shanghai, China). These experiments were run simultaneously, thus the anti-ACE2 mAb served as positive control of binding for all the analysis conducted. The standard S1 subunit used as reference in the study corresponds to SARS-Cov-2 DNA sequence encoding YP_009724390.1, consisting of 681 amino acids (76.5 kDa) with a polyhistidine tag at the C-terminus (Cat: 40591-V08H; Sino Biological).

## 3. Results and Discussion

### 3.1. Design Strategies for Accelerated Expression of SARS-CoV-2 RBD in HEK293SF Suspension Cells

**Figure 1:**
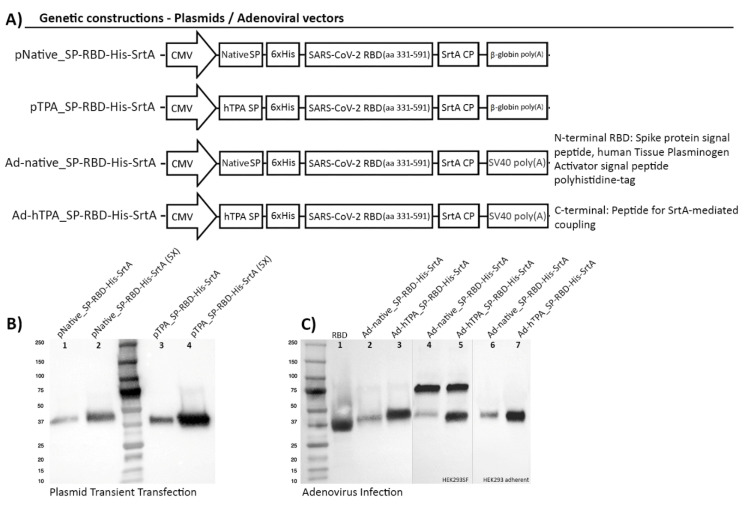

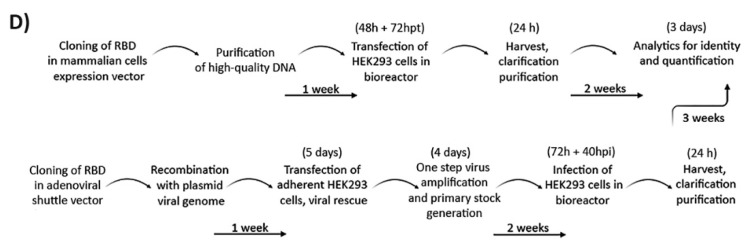
Genetic construction design of SARS-CoV-2 RBD expression cassettes and detection of the recombinant protein by transient transfection and adenoviral infection of HEK293SF cells. (**A**) Schematic representation of constructions encoding the RBD preceded by the native Spike signal peptide (SP) or the human Plasminogen Tissue Activator (hTPA) secretion signal, followed by a polyhistidine tag (6xHis). The coding sequence for the bacterial Sortase A coupling peptide (SrtA, CP) was added at the C-terminal of the protein. The diagram shows the regulatory regions that control the expression of RBD: (i) a fragment of the human cytomegalovirus enhancer/promoter (hCMV) was used in all the constructions, (ii) the rabbit β-globin polyA signal was used in the plasmid constructions for transient transfection, and the SV40 polyA fragment in the adenoviral constructions. (**B**) Detection by Western Blot of RBD expression in 25 mL of culture supernatant 72 h post-transfection (hpt) in shake flasks using HyCell TransFx-H medium. An anti His-tag monoclonal antibody was used. The secreted antigen is detected with an apparent molecular weight of approximately 38 kDa. Lanes 1, 2 correspond to the protein expressed under the native Spike signal peptide and lanes 3, 4 to secretion driven by the hTPA secretion signal. In lanes 2 and 4, (5X) corresponds to a 5-fold concentration of culture supernatant. Equal amounts of total proteins (3.6 ug for lanes 1 and 3 and 12.6 ug for lanes 2 and 4) were loaded onto the gel, enabling to confirm that higher RBD secretion levels were obtained with the use of the hTPA signal peptide. (**C**) Detection by Western blot of RBD expression mediated by adenoviral infection of HEK293SF cells. The anti His-tag monoclonal antibody (lanes 1–3) and a polyclonal anti-SARS-CoV-2 Spike antibody (4–7) were used. The protein was similarly expressed with a molecular weight of about 38 kDa. The polyclonal antibody used showed non-specific binding to one protein identified as lactoferrin, present in the culture medium at a molecular weight of approximately 75 kDa. Lanes 2, 4and 3, 5 show the expression of RBD using the native signal peptide and hTPA, respectively, at the time of harvest, 40 h post-infection, as recognized with each antibody. Lanes 6 and 7 correspond to detection of RBD under the two signal peptides in HEK293 adherent cells at the time of virus rescue (note no presence of lactoferrin at 75 kDa when using DMEM + 10%FBS). Lane 1 in (**C**) corresponds to RBD used as positive control, as referenced in Materials and Methods. (**D**) Diagram representing the workflow and timelines for the accelerated production of RBD following the two methods described.

Four genetic constructions were generated with the aim to achieve RBD production at high levels in an accelerated manner, using an easily scalable system of HEK293SF cells in suspension. An extended segment of the Receptor Binding Domain in the SARS-CoV-2, covering amino acid residues 331 to 591 was selected for expression, producing a recombinant protein of 261 amino acids. RBD extends from amino acid residues 318 to 510 in SARS-CoV [20,21]. However a previous study showed that a CHO-produced recombinant RBD containing these residues could not fully protect vaccinated mice from SARS-CoV challenge, while an extension of 26 residues (until amino acid 536) was able to induce complete protection [22]. The 331–591 RBD segment here selected contains 8 predicted CD4 T cell epitopes and 10 predicted CD8 T cell epitopes in addition to potential B cell epitope motifs [11,12], rendering a potentially more effective immunogen. RBD expression in the present work was driven by the human CMV promoter in all the constructions. Since secretion to the culture medium was critical for an easy recovery of the antigen, the efficiency of the native Spike signal peptide was compared with the hTPA secretion signal, which has been highly successful in the secretion of diverse recombinant proteins [23]. The constructions consisted of one DNA expression plasmid previously optimized for expression in mammalian cells and a shuttle plasmid for the generation of non-replicative human type 5 adenoviruses (evaluating in each case the two different signal peptides). These constructions permitted to evaluate both an efficient rapid transfection methodology of the cells in suspension and an alternative virus-dependent bioprocess amenable for further scale-up and mass production of RBD. The protein was designed to display a coupling peptide (CP, consisting of 6 extra amino acids) at the C-terminal end, with the purpose to be used via a bacterial transpeptidase (Sortase A, SrtA) in the direct attachment, through a covalent bond, of the RBD antigen to more complex high molecular weight protein structures that can function as effective carriers or molecular adjuvants, enhancing the RBD-specific humoral and cell-mediated immune responses in immunized individuals [24,25,26] (Figure 1A). This enzyme-mediated bioconjugation to a protein-based carrier is higher in efficiency compared to other approaches, and occurs with preservation of the proper protein folding. Appropriate folding of RBD with a similar length proved to be able to efficiently bind the ACE2 receptor, as measured by bio-layer interferometry (BLI) [27]. Selecting this larger fragment may also assist the folding into a more stable and immunogenic conformation, with the ability to induce more efficacious protective immunity. Regarding the full-length Spike protein, its use has been associated with antibody-dependent enhancement of disease (ADE) [28]. In rare cases, pathogen-specific antibodies can promote pathology, resulting in the phenomenon known as ADE. In ADE, binding of a virus to suboptimal antibodies could enhance its entry into host cells. Previous studies showed that the immunization with inactivated whole SARS-CoV, modified vaccinia virus Ankara (MVA)-encoded S protein and DNA vaccine encoding full-length S protein could induce ADE or eosinophil-mediated immunopathology, but probably due to low quality and quantity of antibody production [29]. Therefore, it is crucial to determine which vaccines and adjuvants/carriers can elicit protective antibody responses to SARS-CoV-2 [28,29] and implement robust designs and vaccination strategies to guarantee their enhancement.

For RBD expression, small-scale transfections were conducted with PEI at cell densities of 1 and 2 × 10^6^ cells/mL (in HyCell TransFx-H) in order to optimize the operation conditions using HEK293SF cells in suspension. The expression of RBD was confirmed by Western blot analysis, performed with samples of culture supernatant from transfected cells, collected at 72 h post-transfection. RBD expression mediated by the plasmids pNative_SP-RBD-His-SrtA and pTPA_SP-RBD-His-SrtA is shown in Figure 1B, in which the recombinant RBD migrates with an apparent molecular weight of around 38 kDa. Equal amounts of protein were loaded onto the SDS-PAGE gels after identical transfection and culture conditions, thus enabling to identify a superior performance of the hTPA signal peptide over the Spike native sequence (Figure 1B). In these assays, the polyclonal anti-SARS-CoV-2 Spike antibody showed a non-specific binding to one protein identified as lactoferrin (Figure 1C), present in the culture medium, at a molecular weight of approximately 75 kDa. This non-specific binding of various unrelated polyclonal or monoclonal antibodies to lactoferrin has been previously documented in the literature and its identity was previously confirmed by mass spectrometry in our group [30]. In a different approach, RBD production mediated by the adenoviral vectors Ad-native_SP-RBD-His-SrtA and Ad-hTPA_SP-RBD-His-SrtA, both rapidly rescued after a single amplification step in HEK293SF cells, resulted in an identical expression profile, with a major band at approximately 38 kDa. This band was indistinguishable from the previous one obtained by transfection and evidenced as well superior levels of expression under the hTPA signal peptide (Figure 1C). The diagram in Figure 1D summarizes the key steps and timelines of the RBD production using the two transient expression approaches.

### 3.2. Scale-Up for Mass Production in Bioreactors

**Figure 2:**
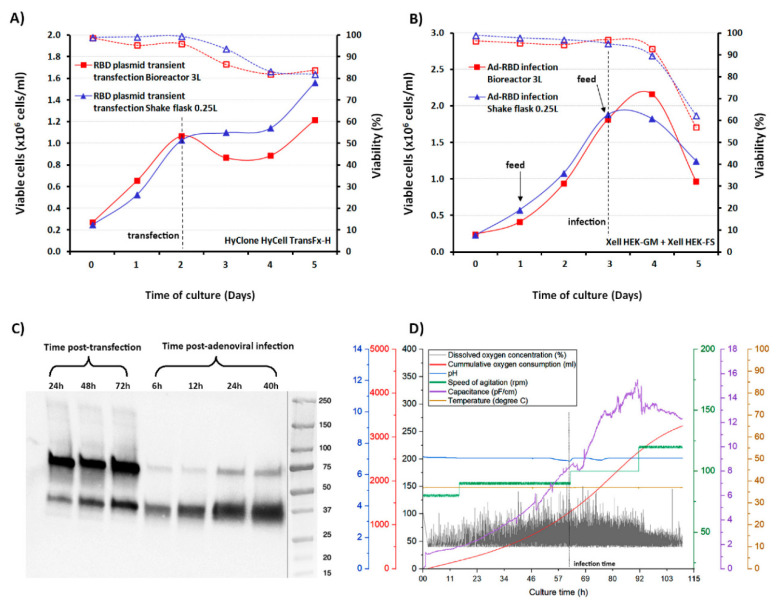
Characterization of RBD expression kinetics in HEK293SF cells grown at small scale in shake-flask and in 3L bioreactors, following plasmid DNA transient transfection or adenoviral infection. (**A**) Time course of the viable cell growth and cell viability of HEK293SF cells cultured in batch mode in HyCell TransFx-H medium, transfected with the plasmid pTPA_SP-RBD-His-SrtA at a cell density of 1 × 10^6^ cells/mL at small scale (250 mL shake flasks) and in 3L-Bioreactor. The time of transfection for both cultures is indicated with an arrow. (**B**) Time course of the viable cell growth and viability of HEK293SF cells in Xell HEK-GM medium for RBD production mediated by Ad-hTPA_SP-RBD-His-SrtA adenovirus infection. The time course of cultures with the same volumes as in (**A**), operated in fed-batch mode are shown, with the feed supplement (Xell HEK-FS) initiated at a cell concentration of 1 × 10^6^ cells/mL and administered every two days by bolus addition at 5% (*v/v*) (indicated in the figure with arrows). The infection was set at a cell density of 2 × 10^6^ cells/mL. The cultures were strictly monitored and harvested when the cell viability reached approximately 70%. (**C**) Time-course expression analysis of the RBD secreted to the culture in the 3L bioreactor productions conducted by transient transfection (24–72 hpt) or adenoviral infection (6–40 hpi) of the cells. The RBD detection was conducted by Western Blot using the polyclonal antibody. (**D**) In-line measurements and profile of various bioreactor sensors and process parameters from the 3L-RBD production mediated by the adenovirus are shown (speed of agitation, pH, dissolved oxygen concentration, cumulative oxygen, and temperature). In addition, the curve of the capacitance values shows the changes registered after infection as the cells undergo the productive phase, in which expression and secretion of the RBD also occurs. The dotted line indicates the time point of infection.

Controlled bioreactor runs of three liters volume were operated in order to evaluate the scalability of the process, via both the non-viral and viral production strategies. For the plasmid DNA transfection, the bioreactor was seeded at 0.25 × 10^6^ cells/mL. In parallel, 2.8 mg of transfection-quality plasmid were purified and used to transfect the HEK293SF culture following the method described. Cell viability deceased to around 80–90% after transfection, and the number of viable cells remained relatively constant during the next 72 hpt, with a slight increase from around 1 to 1.5 × 10^6^ cells/mL (Figure 2A). For adenoviral infection in bioreactors, we took advantage of advances in process development performed by our group for the production of adenoviral vectored vaccines using HyCell TransFx-H and Xell HEK-GM culture media [15]. Previously, supplementing the cells with the medium enriched with feeding additives had an important effect on the production phase of the cultures, significantly increasing the specific virus per cell yield. 

HEK293SF cells were cultured at small scale in each of these media, and infected at 1 and 2 × 10^6^ cells/mL with the Ad-hTPA_SP-RBD-His-SrtA viral vector. The cultures were fed as described in Materials and Methods with the corresponding supplement. Consistently higher yields of RBD were detected (1.5-fold) using the Xell HEK-GM plus HEK-FS combination, based on ELISA determinations (Table 1). Infections at higher cell density (5 × 10^6^ cells/mL), at a MOI = 5 and MOI = 0.1 were also evaluated, with around 2.3-fold higher yield values of RBD also obtained when using the Xell HEK-GM medium at a MOI = 5. In the 3L bioreactor, characteristic adenoviral infection curves were observed when cells were infected with the Ad-hTPA_SP-RBD-His-SrtA viral vector at a cell density of approximately 2 × 10^6^ cells/mL, a MOI of 5, and the HEK-GM basal medium plus feeding. The viable cell count slightly increased after infection and rapidly started to decrease, reaching cell viability values below 70% at around 40 h post-infection (hpi). The monitoring of cell viability in both bioreactors appears in the Figure 2A,B, in which the time of feeding, time of transfection and time of infection are indicated. As depicted in Figure 2C, the kinetics of expression, as analyzed by Western blot, shows the recombinant RBD migrating with an apparent molecular weight of around 38 kDa in the culture supernatant of both production processes. As expected, some differences were observed in the expression pattern of proteins secreted to the culture medium during RBD production. Detection of RBD expression in infected HEK293SF cells occurred as early as 6 hpi. Production yields kept rising, as expected, until 40 hpi. Expression levels in the culture supernatant of transfected or infected cells were determined based on ELISA quantifications. About 7-fold RBD yield increase was found following the viral approach (Table 2). The preparation of a virus seed stock for the Ad-hTPA_SP-RBD-His-SrtA was also undertaken. Clearly the availability of a virus seed stock represents a significant advantage over the transient transfection procedure, which requires repeated laborious amplification/purification rounds to obtain high-quality plasmid DNA for transfection. The 3L bioreactor runs were also in-line monitored for process parameters, including the use of a capacitance probe, whose signal constituted an indicator of the total biovolume of the cultures and a marker of cell physiological changes during the bioprocess. The variations in capacitance and conductivity were also indicators of the total biomass during cell growth in the initial phase of the virus-infected culture, evidencing the cells shift to the viral production phase (Figure 2D). In this second phase, the RBD also starts to be produced and is properly processed for secretion to the culture medium.

### 3.3. Downstream Processing and Characterization of the Antigens

Downstream processing for purification of the extended RBD fragment was conducted similarly for the RBD proteins secreted by the two methods of production. The His tag at the N-terminal of the protein facilitated an efficient purification of the antigen following steps of centrifugation for cell pellet separation, 0.2 μm filtration, buffer exchange and immobilization/elution in a metal affinity chromatography (IMAC) column. Additionally, the protein carries one endopeptidase cleavage site for the His tag removal if required. Buffer exchange became an essential step since Ni-NTA stripping was observed when the culture medium was loaded directly into the HisTrap nickel column. This case of Ni stripping was observed when using the two different media evaluated for production. The RBD could not be detected in the permeate fraction of the buffer exchange step in which centrifugal units with a cut-off of 10 kDa were used. This was confirmed by the ELISA, western blot analyses and quantification of total proteins in the permeate fraction. The buffer exchange retained fraction was then loaded into the Ni-NTA column for purification.

The protein showed high affinity to the column, since negligible RBD amounts were detected in the column flow-through fraction after a maximum of 650 mL of transfection culture supernatant was passed. Washing the column with 60 mM imidazole concentration was found to be sufficient for removal of most impurities, with undetectable RBD being removed from the column, according to the detection methods described. The RBD was eluted at 150 mM imidazole concentration, resulting in purification recoveries over 50% for the RBD produced by the two different methods, when different batches from the 3L productions were processed. A high purity over 95% was estimated by SDS-PAGE and silver staining based on densitometry analysis of the elution fraction. An additional polishing step will be introduced after Sortase-mediated coupling of RBD in a final purification step of the subunit vaccine candidate.

**Figure 3:**
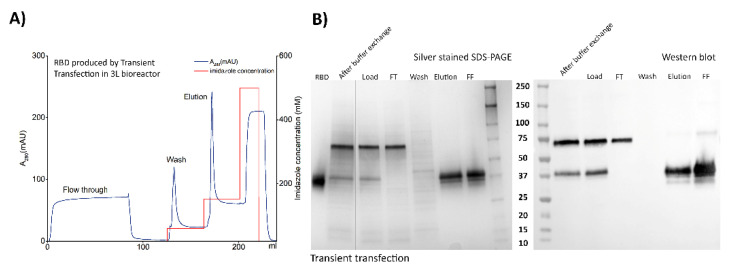

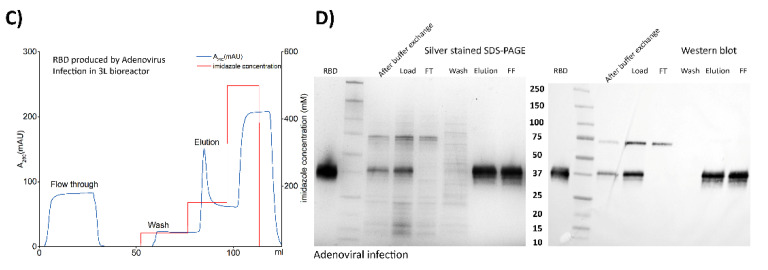
Downstream processing conducted for recovery and purification of the RBD transiently expressed in 3L-controlled bioreactors following two different strategies. (**A**) Chromatogram of IMAC purification of RBD in culture supernatant produced from plasmid DNA transfection of HEK293SF cells. Main purification steps are indicated by UV absorbance at 280 nm and they comprise the flow-through after loading the column, a washing step and elution of purified RBD. (**B**) Analysis by silver stained SDS-PAGE and Western blot using an anti-Spike polyclonal antibody of different fractions from the RBD separation and purification steps, after transient transfection of HEK293SF cells. The samples corresponding to steps or fractions such as buffer exchange (to eliminate the HyCell-TransFx-H medium), loading, flow through (FT), wash, elution and final formulation (FF) appear in both panels in which the RBD can be observed with a high degree of purity at a molecular weight of around 38 kDa. (**C**) Chromatogram of IMAC purification of RBD in culture supernatant produced from adenoviral infection of HEK293SF cells. The main purification steps of RBD are indicated by UV absorbance at 280 nm. (**D**) Silver stained SDS-PAGE and Western blot analysis of samples and fractions collected from an identical purification process as the one described in (**A**,**B**). The molecular weight marker used and reference RBD are also shown in the figure.

The expression pattern of purified RBD showed the wide band migrating at approximately 38 kDa (Figure 3A,B). This band was clearly identifiable by SDS-PAGE in the retentate samples collected after buffer exchange and in the nickel column loading material. It was detected using polyclonal anti-S and monoclonal anti-His antibodies. The non-specific reaction of both the polyclonal and monoclonal antibodies with lactoferrin, as previously explained, was observed in both culture media and also in the purification samples analyzed. This contaminant was detected at a very low concentration in the final eluted samples only by western blot. Its observation was not possible with silver staining, indicating total protein quantities of less than 5–10 ng per 1 µg of RDB.

IMAC purification conditions were identically applied to the RBD produced by the transient transfection approach and adenovirus infection with similar results. An additional ultrafiltration step (300 kDa cut-off) was introduced before the buffer exchange step for the adenovirus-produced RBD, in order to remove the adenovirus particles from the supernatant. Complete removal of adenoviral particles with this step was demonstrated by TCID_50_ titration experiments in which no viral particles could be detected. The expression levels and recovery values of RBD in the purification process are summarized in Table 2, Table 3 and Table 4.

**Figure 4:**
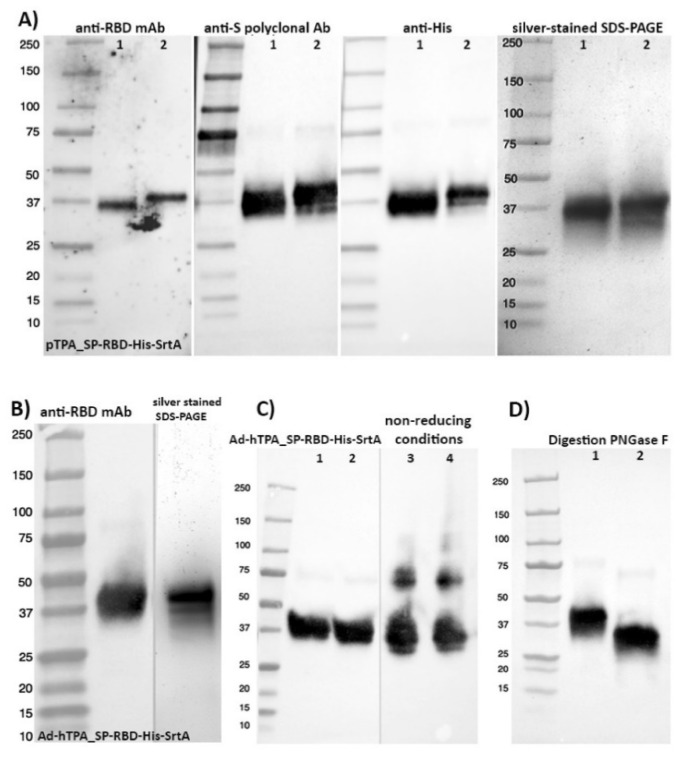
Characterization of final pools of purified RBD obtained in 3L-controlled bioreactor productions. or adenoviral infection. The analysis of RBD obtained by transient transfection (**A**) was performed with monoclonal and polyclonal antibodies against the Spike protein. Lane 1 in all panels shows reference RBD protein. (**B**) RBD from the adenoviral transfected culture. (**C**) Two different batches of RBD were expressed and purified as in (**B**) and were analyzed under reducing (lanes 1, 2) and non-reducing (lanes 3, 4) conditions by western blot using the polyclonal anti-S antibody. Under non-reducing conditions, the detection of high molecular weight structures over 75 and 110 kDa suggests the formation of multimeric structures. (**D**) Western blot, using the anti-S polyclonal antibody, of RBD obtained as in (**B**), before (lane 1) and after (lane 2) enzymatic deglycosylation with PNGase F.

Both antigens (the one obtained by plasmid transfection and the one obtained after adenovirus infection) were detected in western blot analysis with an anti-Spike S1 monoclonal antibody HC2001, after pooling of the batches independently purified (Figure 4A,B). Importantly, the proper conformation of both antigens was demonstrated by ELISA (competitive and direct ELISA) through binding to the anti-RBD CR3022 monoclonal antibody, enabling its quantification with high accuracy in raw, clarified and purified samples. The CR3022 monoclonal antibody is a neutralizing antibody obtained from a convalescent SARS-CoV–infected patient [20,31,32], it targets a highly conserved epitope distal from the receptor binding site, and shows cross-reactive binding between SARS-CoV-2 and SARS-CoV. Structural modeling demonstrated that this binding epitope can only be accessed by CR3022 when at least two RBDs on the trimeric S protein are in the “up” conformation and slightly rotated [31]. The latter suggested a good conformational resemblance to native RBD. In addition, analysis of the 38-kDa RBD under non-reducing conditions suggested the formation of high molecular weight structures, with protein bands detected migrating around 80 and 115 kDa (Figure 4C).

This RBD pool purified from transfected HEK293SF cells was also analyzed by Western blot after enzymatic digestion with PNGase F, showing a product that decreased in molecular weight from 38 to approximately 32 kDa, indicating that the recombinant protein is N-glycosylated (Figure 4D). The monosaccharide and sialic acid content was also determined in the RBD protein from the two methods described and was found to be very similar, as shown in Table 5. The RBD is heavily glycosylated. Both samples showed the presence of monosaccharides consistent with N-glycosylation (Fuc, GlcN, Gal and Man). The additional presence of GalNac in both samples might indicate the presence of O-glycosylation on Ser/Thr [33]. Glucose (Glc) presence in glycoproteins could be due to a misfolded or non-native protein that was labelled with glucose to be reprocessed in the endoplasmic reticulum [34]. In the present case, glucose is likely stemming from the hydrolysis of sucrose traces remaining after purification of the bulk sucrose in the media through the 3 kDa MW filter. Work on N-glycosylation with hydrophilic interaction chromatography (HILIC) and glycopeptide analysis using MS are required to confirm presence of O-glycans and the nature of N-glycans. To date, publications on the glycosylation of the spike protein have shown a wide variation in the glycosylation patterns even when using the same expression system (HEK293 cells). These differences in glycans could also potentially affect the immunogenicity of RBDs [35,36,37,38]. The S protein has multiple glycosylated sites and in silico 3D simulations have predicted the formation of a dense coating on the surface [39], whose role on the protein conformation and immune evasion is still under study. The SARS-Cov-2 surface shows areas of vulnerability despite its dense carbohydrate layer, and since a potent neutralizing antibody binds to a glycosylated epitope, sugars could be less impactful allowing sufficient exposition of peptide epitopes as is the case for the influenza virus [39].

**Figure 5:**
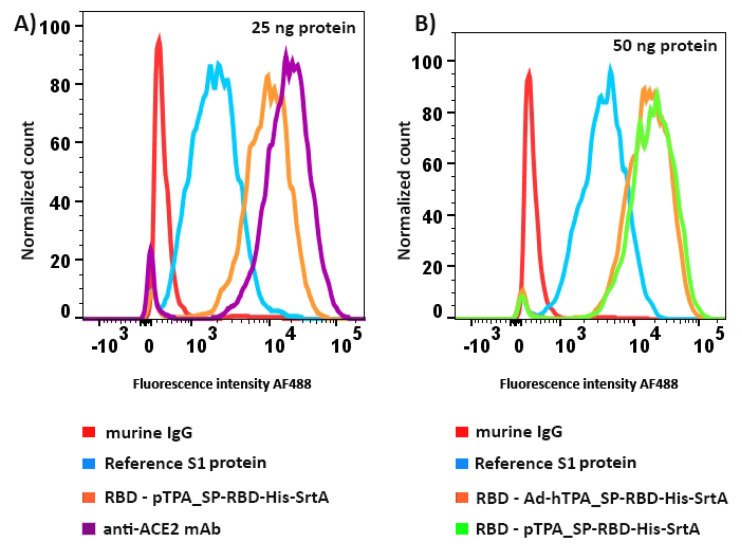
Histograms of the flow cytometer analysis for evaluation of RBD binding to human ACE2 expressed by a stably transfected HEK293 cell line in culture. (**A**) Direct binding assessment of S1 reference protein and highly purified RBD obtained by the transient transfection mode of production in this work. Binding of unrelated murine IgG or the high-affinity of an anti-ACE2 monoclonal antibody is also shown. (**B**) Simultaneous assessment of binding to ACE2 of S1 reference protein and RBD obtained by the two methods of production; transient transfection and adenoviral infection of HEK293SF suspension cells. For detection, an anti-His tag monoclonal antibody conjugated to Alexa Fluor 488 was used. Binding of the anti-ACE2 monoclonal antibody was run as the positive control in the whole experiment. RBD was assayed at a concentration of 0.25 µg/mL (panel A) and 0.5 µg/mL (panel B).

Finally, since ACE2 is a functional receptor for SARS-CoV-2, and it is known that the virus possesses functional gains in attachment, with a greater affinity and enhanced ACE2 recognition compared to SARS-CoV [40], RBDs highly purified (over 95% purity) after the downstream process described were assessed for their direct binding to ACE2 and quantified by flow cytometry. ACE2-expressing HEK293 adherent cells and a monoclonal anti-His tag antibody conjugated to a fluorochrome were used. A strong binding to ACE2 was evidenced at different doses assayed (25 and 50ng), and was found to be very similar between the RBD obtained via the two production methods. Binding to ACE2 was also superior when compared with binding of a recombinant S1 reference protein used as control in the assay and very similar to the binding of an anti-ACE2 monoclonal antibody (Figure 5). Similar binding capacity was also demonstrated for RBD purified from HEK293SF cultures in different production lots.

## 4. Conclusions

Although different types of vaccines against SARS-CoV-2 are in pre-clinical stages or entering Phase 1–3 trials, research and investments in the development of new and existing vaccine platforms are still needed to effectively tackle the impact of the present pandemic and the threat of upcoming global health crises. This work emphasizes the fast-tracked production of an extended segment of SARS-CoV-2 Spike RBD in HEK293SF cells, cultured in suspension, by two different bioprocesses. The non-viral and viral production approaches developed, consisting on plasmid transfection or adenoviral infection of HEK293SF cells, achieved the proposed goal of generating high yields of a functional recombinant RBD product following simple, scalable, upstream and downstream protocols, with added features to increase its immunogenicity such as the ability of coupling to a carrier protein. Some of the vaccines presently in development are S-based subunit vaccines or their variations, including the putative use of these molecules as a tool for the design and testing of blocking or inhibitor therapeutic agents. However, obtaining the Spike protein or its subunits with high resemblance to the wild-type viral protein, as they are synthesized in the human host, is crucial for protective immunity and related purposes. One advantage of HEK293SF cells as expression system is related to the achievement of proper post-translational modifications, specifically glycosylation, which could be rather inadequate or has unexpected implications for vaccine efficacy if produced in non-human cell lines.

The main monoclonal antibody used in this study for RBD quantification and characterization binds to an epitope conserved between SARS-CoV-2 and SARS-CoV that is distinct from the ACE2 receptor-binding site. As mentioned, structural modeling shows that this epitope is only revealed when at least two of the three spike proteins are in a conformation competent to bind the receptor. The latter strengthens our assumption of a truly native-like conformation of the RBD produced, since it provides evidence on two critical regions properly folded; the ACE2 binding site and the CR3022 mAb binding epitope. This work showed the RBD binding capacity produced by the two bioprocesses described. RBD can be successfully expressed and recovered from a variety of expression systems; however its antigenicity and the ability to effectively bind its cell receptor implies more complex requirements influenced by factors such as the system of expression, culture conditions, downstream processing steps and stability both throughout the whole process and in the final formulation.

The strategies described in this study do not limit themselves to the production of RBD. The overall process design can be used in a flexible mode to express a full-length spike protein, one of its subunits, or a multi-epitopic construct strategically designed for an enhanced immunogenicity and protective efficacy. These platforms, and specially the adenoviral-based approach, can produce the desired antigen within a short span of time and allows to be operated at larger scale for the rapid generation of the antigen for its further evaluation in pre-clinical and clinical trials. Finally, the RBD here produced carries a sortase coupling domain, which has been shown to be an effective instrument for the coupling of full-length viral proteins to protein-based carriers such as virus-like particles or nanoparticles, with the aim to achieve an improved, more versatile immune response in vaccinated individuals. Using this tool is the next rational step within this bioprocess for production of a RBD-based subunit vaccine against SARS-CoV-2.

## Figures and Tables

**Table 1:** RBD expression levels determined by ELISA in the supernatant of HEK293SF cells cultured under different conditions in suspension and serum-free media.

Sample	Culture Conditions *	Cell Density at Transfection/Infection	Sampling Time	RBD—ELISA (µg/mL)
Culture supernatant from adenovirally infected HEK293SF cells	Shake flasks, HEK-GM + HEK-FS	2 × 10^6^ cells/mL	40 hpi	115.3 ± 6.2
		1 × 10^6^ cells/mL		93.4 ± 17.6
	Shake flasks, HyCell-TransFx-H + Cell Boost 5	2 × 10^6^ cells/mL	40 hpi	82.2 ± 3.0
		1 × 10^6^ cells/mL		62.6 ± 7.3
Culture supernatant from HEK293SF cells adenovirally infected at high cell density	Shake flasks, HEK-GM + HEK-FS	5 × 10^6^ cells/mL	40 hpi	67.5 ± 34.5
	Shake flasks, HyCell-TransFx-H + Cell Boost 5	5 × 10^6^ cells/mL		28.4 ± 4.5
Culture supernatant from transfected HEK293SF cells	Shake flasks HyCell-TransFx-H	1 × 10^6^ cells/mL	24 hpt	1.5 ± 0.23
			48 hpt	5.8 ± 0.7
			72 hpt	17.7 ± 5.1
Culture supernatant from adenovirally infected HEK293SF cells	Shake flasks Xell HEK-GM + HEK-FS	2 × 10^6^ cells/mL	6 hpi	15.3 ± 1.1
			24 hpi	62.2 ± 19.3
			40 hpi	131.7 ± 27.2

* Adenovirus MOI = 5 was used in all the shake flask experiments.

**Table 2:** RBD expression levels determined by ELISA in the supernatant of HEK293SF cells cultured in 3L bioreactors for production of the recombinant SARS-CoV-2 Spike RBD.

Sample	Culture Conditions	Cell Density at Transfection/Infection	Sampling Time	RBD—ELISA (µg/mL)
Bioreactor harvest broth from transfected HEK293SF cells	3L-bioreactor operated in batch mode (HyCell-TransFx-H medium)	1 × 10^6^ cells/mL	72 hpt	17.8
Bioreactor harvest broth from adenovirally infected HEK293SF cells	3L-bioreactor operated in fed-batch mode (Xell HEK-GM medium + HEK-FS supplement)	2 × 10^6^ cells/mL (MOI = 5)	40 hpi	101.7

**Table 3:** RBD expression levels and recovery determined by ELISA in samples collected during RBD purification from the supernatant of transfected HEK293SF cells cultured in suspension in a 3L bioreactor.

RBD by Plasmid Transient Transfection 3L Bioreactor	Total Protein Concentration (µg/mL)	RBD—ELISA (µg/mL)	Volume (mL)	Purification Recovery (%)
Ni-NTA Load after harvest broth centrifugation, 0.2 µm filtration, medium exchange using 10 kDa cut-off	345.4	34.4	118	100
Ni-NTA Column flow-through	231	below L.O.D *.	118	-
Wash	7	below L.O.D.	10	-
Elution	152	158.8	15	58.6

* LOD: Limit of detection.

**Table 4:** RBD expression levels and recovery determined by ELISA in samples collected during RBD purification from the supernatant of adenovirus infected HEK293SF cells cultured in suspension in a 3L bioreactor.

RBD by Adenovirus Infection 3L Bioreactor	Total Protein Concentration (µg/mL)	RBD—ELISA (µg/mL)	Volume (ml)	Purification Recovery (%)
Ni-NTA Load after harvest broth centrifugation, ultrafiltration 300 kDa, medium exchange 10 kDa	392.5	112.5	26	100
Ni-NTA Column flow-through	212.8	8.1	26	7.2
Wash	8.4	below L.O.D *.	10	-
Elution	129.8	104.4	15	53.5

* LOD: Limit of detection.

**Table 5:** Monosaccharide and sialic acid molecular ratio (Mol Monosaccharide/Mol RBD) after hydrolysis or enzymatic release for RBD produced via a viral and a non-viral transient expression vector in HEK293SF cells, cultured in suspension and serum-free media in 3L controlled bioreactors.

Monosaccharide	RBD by Adenoviral Infection	RBD by Transient Transfection
Fuc	1.0	1.2
GalN	0.8	1.2
GlcN	4.2	4.2
Gal	1.6	1.3
Glc	13.1	5.7
Man	2.9	2.7
Sialic acid	2.0	1.6
